# Behavioral Therapy as an Adjunct to Buprenorphine Treatment for Opioid Use Disorder: A Secondary Analysis of 4 Randomized Clinical Trials

**DOI:** 10.1001/jamanetworkopen.2025.28529

**Published:** 2025-08-20

**Authors:** R. Kathryn McHugh, Allen J. Bailey, Brooke A. McConaghy, Roger D. Weiss, David A. Fiellin, Maureen Hillhouse, Brent A. Moore, Garrett M. Fitzmaurice

**Affiliations:** 1 Division of Alcohol, Drugs and Addiction, McLean Hospital, Belmont, Massachusetts; 2 Department of Psychiatry, Harvard Medical School, Cambridge Massachusetts; 3 Department of General Internal Medicine, Yale School of Medicine, New Haven, Connecticut; 4 Marron Institute, New York University, Brooklyn, New York; 5 Department of Psychiatry, Yale School of Medicine, New Haven, Connecticut

## Abstract

**Importance:**

Several large, randomized clinical trials have tested the efficacy of adding behavioral therapy to medical management (high-quality, low-intensity medical counseling) and buprenorphine treatment of opioid use disorder. These studies have consistently reported strong rates of treatment response overall, without a significant additive benefit of additional behavioral therapy.

**Objective:**

To address gaps in knowledge about additional behavioral therapy for patients receiving buprenorphine, including the association of additional behavioral therapy with retention and functional outcomes, and whether certain subgroups respond better to additional behavioral therapy.

**Design, Setting, and Participants:**

This study is a secondary analysis of 4 randomized clinical trials conducted in Connecticut, Southern California, and 10 other US sites between 2000 and 2011. Participants included adults with *Diagnostic and Statistical Manual of Mental Disorders (Fourth Edition)* opioid dependence. Analyses were conducted between January 2024 and July 2025.

**Exposure:**

Buprenorphine and varying levels of behavioral therapy, including standard medical management, physician management, physician management plus cognitive behavioral therapy, contingency management, contingency management plus cognitive behavioral therapy, standard medical management plus opioid dependence counseling, or no additional behavioral treatment.

**Main Outcomes and Measures:**

The main outcomes included weeks of buprenorphine retention and functioning across 7 domains (medical, employment and financial support, social and family, alcohol, drug, legal, and psychiatric), assessed using the Addiction Severity Index. Data on additional behavioral therapy (structured cognitive-behavioral and counseling approaches) combined with buprenorphine and medical management were harmonized to provide needed statistical power for considering moderation effects.

**Results:**

The combined sample consisted of 869 adults (mean [SD] age, 34.2 [10.4] years; 287 female [33%]). Results demonstrated that additional behavioral therapy was not associated with opioid-free weeks (mean [SD] number of opioid-free weeks, 7.16 [4.35]) compared with medical management and buprenorphine (mean [SD] number of opioid-free weeks, 7.00 [4.33]) (B = 0.28; 95% CI, −0.33 to 0.89; *P* = .37). Additional behavioral therapy was also not associated with greater buprenorphine retention (mean [SD] number of weeks of buprenorphine, 10.29 [3.21] out of 12) compared with medical management and buprenorphine (mean [SD] number of weeks of buprenorphine, 10.21 [3.15]) (B = 0.00; 95% CI, −0.43 to 0.43; *P* = .98). Measures of functioning indicated minimal change over the course of treatment, and there were no differences between randomized groups. No moderational effects of subgroups (eg, history of heroin use) were significant when correcting for multiple comparisons.

**Conclusions and Relevance:**

In this secondary analysis of 4 randomized clinical trials, results highlighted the strong efficacy of buprenorphine treatment when combined with medical management for opioid use disorder. Although there was certainly room for improvement in outcomes—particularly functioning—trials of novel adjuncts for buprenorphine treatment may encounter statistical power challenges outperforming such a robust control condition.

**Trial Registration:**

NCT00316277, NCT00591617, NCT00632151, NCT00023283

## Key Points

**Question:**

What outcomes are associated with additional behavioral therapy in people with opioid use disorder receiving buprenorphine maintenance with medical management?

**Findings:**

In this secondary analysis of 4 randomized clinical trials including 869 adults with opioid use disorder, results indicated that additional behavioral therapy was not associated with additive benefit for treatment retention or functioning outcomes. No significant moderating effects were detected.

**Meaning:**

These findings suggest that buprenorphine plus high-quality, low-intensity medical counseling is highly effective for opioid use disorder.

## Teaser

This secondary analysis of 4 randomized clinical trials whether adding behavioral therapy to buprenorphine is associated with improved treatment retention and functional outcomes among adults with opioid use disorder.

## Introduction

Buprenorphine combined with low-intensity psychosocial support is a highly effective treatment for opioid use disorder (OUD).^1,2,3,4^ Studies of buprenorphine combined with low-intensity, high-quality, manualized medical counseling sessions (ie, medical management [MM]) indicate high rates of abstinence or near abstinence^1,5,6^ and substantial reductions in days of opioid use.^7^ Nonetheless, many people with OUD do not fully respond to buprenorphine with MM, and novel interventions are needed.

Adding high-intensity (eg, weekly) behavioral treatments such as drug counseling^8,9^ or cognitive-behavioral therapy^10,11,12^ to medication has been shown to improve substance use disorder outcomes (eg, naltrexone for alcohol use disorder^13^ and methadone for OUD^14^). In contrast, although some small studies have shown additive benefits of behavioral therapy combined with buprenorphine for reducing opioid use,^15^ large, randomized trials have not shown additive benefits compared with buprenorphine plus MM.^1,5,6^

Several gaps in knowledge about the role of additional behavioral therapy in buprenorphine treatment remain.^16,17^ Most large clinical trials focused on opioid use outcomes, leaving the effects of behavioral therapy on outcomes such as retention and functioning less well known. Additionally, because buprenorphine is highly effective, particularly when combined with MM, it is possible that many people respond adequately to buprenorphine plus MM alone, while others would benefit from additional behavioral therapy.^18,19^ For example, in a prior study,^20^ only 36% of people with co-occurring posttraumatic stress disorder (PTSD) responded to buprenorphine with MM, whereas 67% responded to the combination of buprenorphine, MM, and additional individual drug counseling.

We aimed to fill these gaps using data harmonized from 4 randomized clinical trials^1,4,5,6^ of buprenorphine plus MM with or without additional behavioral therapy.^21^ Building from a previously published review,^17^ our aim was to leverage these existing datasets to consider nonopioid outcomes and moderational effects. Our first aim was to determine whether the addition of behavioral therapy to buprenorphine plus MM would yield improvements in buprenorphine retention or functioning. We hypothesized that behavioral therapy would lead to more weeks of buprenorphine receipt and greater improvement in functional outcomes. Our second aim was to identify whether any subgroups benefited more from additional behavioral therapy by testing moderational effects of demographic and baseline clinical variables on outcomes. Based on prior literature,^18,19,22^ we hypothesized that heroin use, cocaine use, and pain would act as moderators. Other hypothesized moderators included co-occurring psychiatric disorders and symptoms (depression symptoms and PTSD diagnosis), age, functional impairment, and prior treatment episodes.^20,23,24^

## Methods

This secondary analysis was determined exempt by the Mass General Brigham institutional review board. A description of the study rationale and methods have been published previously,^21^ and the data analytic plan was preregistered at Open Science Framework.

### Included Studies

Four clinical trials^1,4,5,6^ were selected for inclusion in this data harmonization trial, based on the use of similar study designs (eg, randomization to a protocolized behavioral therapy) and common data elements. This included 3 National Institute on Drug Abuse–funded R01 trials^4,5,6^ and 1 trial conducted as part of the National Institute on Drug Abuse Clinical Trials Network.^1^ The studies were conducted between 2000 and 2011, and harmonized data were analyzed in July 2024 to January 2025. Data were collected in Connecticut (2 studies^4,6^), Southern California (1 study^5^), and 10 other sites across the US (1 study^1^). Participants in all studies provided written informed consent. All 4 studies collected demographic information on race via self-report. Race categories included Asian, American Indian or Alaska Native, Black or African American, multiracial, Pacific Islander, White, and other (ie, any race not otherwise specified) or not listed. Readers are directed to the primary studies for further trial detail (eg, detailed methods and protocol information and Consolidated Standards of Reporting Trials diagrams).^1,4,5,6^

Fiellin et al^6^ conducted a 24-week trial in which 166 adults with OUD received up to 24 mg of buprenorphine and were randomly assigned to either standard MM (SMM) once weekly, SMM thrice-weekly, or enhanced medical management thrice-weekly. SMM was provided by trained nurses and consisted of manualized, 20-minute weekly drug counseling sessions. Enhanced MM was provided by the same nurses and consisted of similar, yet more in-depth drug counseling in 45-minute weekly sessions.

In a similar study, Fiellin et al^4^ randomized 141 adults with OUD to receive up to 24 mg of buprenorphine, with either physician management or physician management plus cognitive behavioral therapy (CBT). Physician management consisted of 15- to 20-minute weekly, biweekly, then monthly sessions. CBT was provided by master’s level therapists and included 12 weekly, 50-minute sessions.^25^

Ling et al^5^ conducted a 16-week study, in which 202 participants received up to 24 mg of buprenorphine and were assigned to 1 of 4 treatment groups: CBT, contingency management, CBT and contingency management, or no additional behavioral treatment. Therapists administered manualized CBT or CBT and contingency management for 45 minutes each week. Those in the no additional behavior treatment group received limited counseling from study physicians, focused primarily on medical aspects of drug adherence and dosing.

Weiss et al^1^ conducted a multisite trial in which 653 individuals diagnosed with prescription OUD were randomized to either standard SMM alone or SMM plus opioid dependence counseling. The trial was comprised of a 2-phase adaptive, sequential design in which phase 1 of treatment included 2 weeks of buprenorphine with a 2-week taper and 8-week postmedication follow-up. Those who displayed successful opioid use treatment outcomes after phase 1 (43 of 653 participants) were considered successful study completers, whereas those who returned to opioid use were invited to enter extended treatment in phase 2. Only phase 2 data (360 participants) were included in this study. Participants received 12 weeks of stabilization with buprenorphine followed by a 4-week taper. SMM was administered by physicians in weekly 15- to 20-minute sessions. Opioid dependence counseling was delivered in 45-minute sessions and included components of evidence-based counseling approaches.

### Outcomes and Measures

Our outcomes were weeks of opioid use during 12 weeks of treatment, weeks during the 12 weeks of treatment in which buprenorphine was received, and interviewer-rated functioning. Opioid use was assessed using the Timeline Follow-Back Method,^26^ confirmed via urine drug screen. Any week in which opioid use was either self-reported or detected in urine was coded as an opioid-positive week. Retention was defined as a count of weeks in which buprenorphine was received. Functioning was assessed in all studies using the Addiction Severity Index (ASI).^27^ The ASI is an interviewer-administered measure of substance use and functioning, which provides scores across 7 domains: medical, employment and financial support, social and family, alcohol, drug, legal, and psychiatric. ASI scale scores are calculated for each domain and have a possible range of 0 to 1, with higher scores reflecting greater problem severity.

Potential moderators of treatment included age, sex, pain, history of any heroin use (vs prescription opioid misuse only), cocaine use at treatment entry, number of prior treatment episodes, and presence of co-occurring psychiatric disorders (depressive symptoms and PTSD diagnosis). Pain was assessed using the bodily pain subscale of the Short Form 36 Survey.^28^ Lifetime heroin use was a self-reported binary variable assessing whether the patient had ever used heroin in their lifetime. Cocaine use was a self-reported binary variable assessing whether the patient had reported any cocaine use in the 30 days prior to baseline assessment. Depression was not assessed consistently across the 4 studies, with 2 studies^1,5^ utilizing the Beck Depression Inventory (BDI),^29^ and 2 studies^4,6^ utilizing the Center for Epidemiologic Studies of Depression Scale.^30^ Consistent with our preregistration, we conducted sensitivity analyses using the Patient-Reported Outcomes Measurement System depression metric, which allowed for conversion of the 2 distinct depression measures into 1 score, rather than analyzing these scores separately.^34^ PTSD diagnosis was available for 2 of the studies^1,5^ and analyzed as a binary variable indicating PTSD in the last year, obtained from a semistructured diagnostic interview. Studies varied in their inclusion of people with co-occurring psychiatric disorders and there was not sufficient measurement of other psychiatric variables for harmonization.

### Harmonization Process

Although included studies were selected due to their similar research designs, the studies were not specifically designed to be combined, and therefore harmonization required meticulous analysis of data elements across studies. This included careful examination of existing data elements, recoding of variables, and dataset quality control.

### Statistical Analysis

All analyses were completed in R statistical software 4.3.3 (R Project for Statistical Computing)^31^ including use of the mice package for multiple imputation^32^ and the mass package^33^ for count model generalized linear models. As in any longitudinal study, there were missing data, which were minimal for variables collected at baseline (<5%) and largest for posttreatment ASI scores (32%-35%). Opioid use and retention outcomes were examined using a linear count model with identity link function for weeks of opioid use and weeks of buprenorphine received, respectively. See the previously published methods paper for additional detail.^21^ Because there was some variation in the duration of the treatment across studies, we used the first 12 weeks of treatment as our primary end point for these analyses. Examination of moderators was conducted using interaction between randomized treatment condition and each of the 8 moderators in separate linear count models. The α level was set at .01 to adjust for multiple tests. The effect of additional behavioral therapy on functioning was tested using a series of analysis of covariance models, with each ASI score as the outcome; treatment group and baseline level of the ASI score were covariates and α was set at 0.01 to adjust for multiple tests. Moderational analyses considered the interaction between treatment and each moderator of interest on each ASI score. All analyses included an indicator for study and randomized condition and used an intention-to-treat approach. Main and interaction effects of sex were included in all models and all tests were 2-sided.

## Results

The combined sample consisted of 869 participants (287 female [33%]). The mean (SD) age was 34.2 (10.4) years, and self-reported race included 11 American Indian or Alaska Native individuals (1.3%), 11 Asian individuals (1.3%), 56 Black or African American individuals (6.5%), 21 multiracial individuals (2.4%) 21 Pacific Islander individuals (2.4%), 716 White individuals (82%), and 27 individuals with other or not listed race (3.1%). The mean (SD) years of education was 12.8 (2.1) years and X (48.9%) was employed full-time.

### Association of Additional Behavioral Therapy With Clinical Outcomes

Consistent with prior analyses of the included trials using binary outcome definitions,^1,6^ there was no significant association of additional behavioral treatment with opioid-free weeks (B = 0.28; 95% CI, −0.33 to 0.89; *P* = .37) (Table 1). The additional behavioral therapy group had a mean (SD) of 7.16 (4.35) opioid-free weeks out of 12 possible weeks, compared with 7.00 (4.33) weeks for those who did not receive additional behavioral therapy.

**Table 1.** Regression Estimates for Illicit Opioid-Free Weeks

Parameter	B (95% CI)	*P* value
Behavior therapy^a^	0.28 (−0.33 to 0.89)	.37
Study		
Weiss et al, 2011^1^	0 [Reference]	NA
Ling et al, 2013^5^	−1.23 (−2.01 to −0.45)	.002
Fiellen et al, 2006^6^	−0.76 (−1.58 to −0.06)	.07
Fiellen et al, 2013^4^	−1.44 (−2.28 to −0.60)	<.001
Moderators of behavioral treatment (interaction with treatment condition)		
Age	0.02 (−0.04 to 0.08)	.49
Sex (female)	0.56 (−1.81 to 0.69)	.38
Heroin	−0.53 (−1.69 to 0.62)	.37
Cocaine	−1.18 (−2.56 to 0.20)	.10
Prior treatment episodes	−0.09 (−0.22 to 0.04)	.18
Pain	0.20 (−0.07 to 0.47)	.15
Posttraumatic stress disorder^b^	2.33 (0.18 to 4.48)	.04
Depression (Beck Depression Inventory)^b^	0.02 (−0.04 to 0.08)	.56
Depression (Center for Epidemiologic Studies Depression Scale)^c^	−0.03 (−0.13 to 0.07)	.53

Abbreviation: NA, not applicable.

^a^ Behavior therapy parameter is the estimate associated with the addition of behavior therapy compared with buprenorphine plus medical management alone without behavior therapy.

^b^ Measure was only available in Weiss et al^1^ and Ling et al.^5^

^c^ Measures only available in Fiellin et al (2006)^6^ and Fiellin et al (2013).^4^ Moderator estimates were obtained from separate regressions testing the interaction of behavioral therapy and each individual variable.

Additional behavioral therapy was also not associated with retention, defined as weeks of buprenorphine received (B = 0.00; 95% CI, −0.43 to 0.43; *P* = .98) (Table 2). The additional behavioral therapy group received a mean (SD) of 10.29 (3.21) weeks of buprenorphine (out of a possible 12) compared with 10.21 (3.15) weeks in the buprenorphine plus MM only group.

**Table 2.** Regression Estimates on Weeks Receiving Buprenorphine

Parameter	B (95% CI)	*P* value
Behavior therapy^a^	0.00 (−0.43 to 0.43)	.99
Study		
Weiss et al, 2011^1^	0 [Reference]	NA
Ling et al, 2013^5^	−0.74 (−1.29 to −0.19)	.008
Fiellen et al, 2006^6^	−1.62 (−2.27 to −0.98)	<.001
Fiellen et al, 2013^4^	−0.61 (−1.15 to −0.07)	.03
Moderators of behavioral treatment (interaction with treatment condition)		
Age	0.00 (−0.04 to 0.04)	.91
Sex (female)	0.14 (−0.78 to 1.06)	.77
Heroin	−0.44 (−1.26 to 0.38)	.19
Cocaine	−1.04 (−2.06 to −0.02)	.04
Prior treatment episodes	0.01 (−0.09 to 0.07)	.84
Pain	0.17 (0.00 to 0.34)	.049
Posttraumatic stress disorder^b^	0.47 (−1.23 to 2.17)	.59
Depression (Beck Depression Inventory)^b^	0.00 (−0.04 to 0.04)	.93
Depression (Center for Epidemiologic Studies Depression Scale)^c^	−0.05 (−0.13 to 0.03)	.18

Abbreviation: NA, not applicable.

^a^ Behavior therapy parameter is the estimate associated with the addition of behavior therapy compared with buprenorphine treatment plus medical management alone.

^b^ Measure was only available in Weiss et al^1^ and Ling et al.^5^

^c^ Measures only available in Fiellin et al (2006)^6^ and Fiellin et al (2013).^4^ Moderator estimates were obtained from separate regressions testing the interaction of behavioral therapy and each individual variable.

In analyses examining changes in functioning, overall, there were only modest changes in functioning as measured by the ASI from baseline to the end of the trial (follow-up). ASI functioning scores can be seen in Table 3. When correcting for multiple tests, additional behavioral therapy was not associated with differential improvement in any functional domain when correcting for multiple tests (α = 0.01), including alcohol (B = 0.00; 95% CI, −0.02 to 0.01; *P* = .48), drug (B = −0.01; 95% CI, −0.03 to 0.01; *P* = .24), family (B = 0.00; 95% CI, −0.03 to 0.03; *P* = .98), legal (B = −0.01; 95% CI, −0.02 to 0.01; *P* = .55), employment (B = −0.02; 95% CI, −0.06 to 0.02; *P* = .37), medical (B = −0.01; 95% CI, −0.06 to 0.04; *P* = .64), or psychological functioning (B = 0.04; 95% CI, 0.01 to 0.07; *P* = .01). Sex was not associated with treatment outcome in any analyses.

**Table 3.** ASI Functioning Scores Over Time^a^

ASI Domain	ASI functioning score, Mean (SD)	
	Baseline	Follow-up
Drugs	0.28 (0.09)	0.14 (0.12)
Alcohol	0.05 (0.09)	0.05 (0.09)
Family and social	0.13 (0.19)	0.10 (0.17)
Employment	0.41 (0.30)	0.38 (0.30)
Legal	0.06 (0.14)	0.04 (0.11)
Medical	0.17 (0.28)	0.22 (0.31)
Psychiatric	0.15 (0.18)	0.15 (0.19)

Abbreviation: ASI, Addiction Severity Index.

^a^ There were no significant effects of behavioral therapy on any ASI domain.

### Moderational Analyses

We then tested whether any of our a priori moderators influenced the effect of treatment on each of our outcomes. Results of moderational effects on opioid use are presented in Table 1. When correcting for multiple tests, the effectiveness of the additional behavioral therapy in reducing weeks of illicit opioid use was not significantly moderated by age, female sex, lifetime heroin use, cocaine use, prior treatment episodes, pain, PTSD, or depression as measured by the BDI or the CESD. As in the primary analyses, depression was not found to moderate the effect of behavioral therapy for any outcome of interest.

The association of additional behavioral therapy with weeks receiving buprenorphine is presented in Table 2. This association was not significantly moderated by age, female sex, lifetime heroin use, cocaine use, prior treatment episodes, pain, PTSD, or depression as measured by the BDI or the CESD. Similarly, there were no significant moderational effects of any of our hypothesized moderators on any of the ASI domains. 

## Discussion

Due to the impact of OUD on quality of life, functioning, and mortality risk, efforts to optimize treatment response are of critical importance. This secondary analysis harmonized data from 4 randomized controlled trials testing the effects of additional structured, high-intensity behavioral therapies in people receiving buprenorphine plus medical management. The behavioral therapies were structured behavioral and counseling approaches (CBT and individual drug counseling) administered by professional clinicians.

Results suggest that additional behavioral therapy did not yield additive benefits for weeks of opioid use or buprenorphine retention. Of note, across the entire sample, mean rates of opioid-free weeks (>7 out of 12) and weeks of buprenorphine retention (>10 out of 12) were quite high, which may have limited the ability to detect additive benefits. Indeed, Carroll et al^17^ previously posited that the absence of consistent evidence for an additive effect of behavioral therapy may be attributable to the efficacy of buprenorphine, particularly when combined with MM. Although the efficacy of MM has not been systematically studied, they posited that the robust MM used in clinical trials might be sufficient for many people to achieve reduction in opioid use. These MM interventions might be more accurately termed medical counseling because they consisted of many elements of evidence-based counseling interventions, administered at a lower frequency. This effectively rendered these studies a comparison between low- and high-intensity behavioral therapies rather than a comparison between buprenorphine alone and buprenorphine with behavioral therapy. In a recent analysis of these same clinical trial data,^7^ buprenorphine combined with MM resulted in large reductions in opioid use, which substantially limits statistical power for studies of adjunctive interventions. Notably, the MM provided in these studies is unlikely to be consistent with typical care. Indeed, a prior study^2^ of claims data found that found that almost three-fourths of people did not receive any psychosocial services, and that low intensity counseling outperformed buprenorphine alone.

Additional behavioral therapy also did not yield additive benefits for functional outcomes. This result must be considered in light of the very modest improvement in functional outcomes in this sample as well as measurement limitations. Despite its widespread use historically in clinical trials, the ASI has substantial psychometric weaknesses,^35^ including weaknesses in detecting clinical change. It is also possible that functional outcomes truly did not improve in this population, even after at least 18 weeks from the start of treatment. Functional outcomes have been critiqued as a poor marker of treatment-related changes because they are often quite distal from changes in opioid use.^36^ The expectation that potentially long-standing functional domains, such as employment, would change in less than a year may be unrealistic, particularly for people with severe OUD. Finally, it is possible that buprenorphine treatment with MM, even when combined with high-intensity behavioral therapy, may not be sufficient to achieve functional improvement. Targeted attention to factors such as social determinants of health may be needed for functional recovery.

Finally, moderational analyses did not indicate any clear pattern of subgroups that differentially benefitted from the addition of behavioral therapy. A prior study^20^ (using data from 1 of the trials included in this harmonization study^1^) demonstrated a moderational effect of PTSD diagnosis on outcome, such that people with PTSD had a beneficial effect of additional behavioral therapy. In this study, this effect did not reach statistical significance in our adjustment for multiplicity; this may have been attributable to the small sample of people with PTSD in this study, which resulted in the inability to detect small to moderate effects. Further studies may benefit from consideration of treatment response in people with PTSD. In the included studies, only depressive symptoms and PTSD were measured, which precluded the ability to test effects of other psychiatric disorders. Because there is some evidence for a differential influence of psychiatric disorders on retention,^37^ consideration of a greater breadth of psychiatric disorders is an important future research direction. Critically, these results should not be interpreted to suggest that the addition of behavioral therapy targeting the co-occurring disorder would not impact outcomes. The study of adjunctive behavioral treatments specifically for the co-occurring disorder (eg, treatment for PTSD) is a much-needed research area.

### Limitations

This study has limitations. As with any analysis of harmonized study data, although the studies included in our study were carefully chosen to ensure similarities in core data elements and design, they varied somewhat in their treatment duration, dosing, and types of behavioral therapy. To mitigate this weakness, we accounted for each study in all analyses and conducted sensitivity analyses to confirm the robustness of our results to these design features. Another area of heterogeneity was the exclusion in some studies of certain types of polysubstance use (eg, benzodiazepine misuse) and co-occurring psychiatric disorders (eg, major depression and psychosis). Further studies of more heterogeneous samples will be needed to confirm results. In our analyses, we focused on linear effects; consideration of nonlinear effects may elucidate new patterns of responding that can help to inform understanding of additional behavioral therapy. Although this study had more statistical power than individual trials, larger trials may be needed to detect small effects. Additionally, both the drug supply and the treatment landscape for OUD continue to evolve; because these studies were conducted prior to the drastic rise in fentanyl and fentanyl analogues in the drug supply as well as the introduction of new injectable buprenorphine formulations, replication of these results will be needed.

These results should be considered in light of the strong treatment response in the robust comparison condition (buprenorphine treatment plus MM). Importantly, such robust effects suggest that the continued dissemination of buprenorphine treatment with high-quality medical counseling can help many people with OUD to reduce or stop opioid use. Furthermore, the behavioral therapies tested included structured cognitive-behavioral and counseling treatments, and results cannot be assumed to generalize to other behavioral modalities, such as mindfulness-based therapies or other forms of psychosocial support (eg, intensive case management, collaborative care). Such modalities have shown promise^38,39^ and will be important for future study.

### Conclusions

Results of this secondary analysis of 4 randomized clinical trials highlight the efficacy of buprenorphine treatment when combined with medical management for opioid use disorder. Ongoing large-scale clinical trials of treatment for OUD will provide further important information about optimizing treatment for people with OUD. Involving people with lived experience in treatment development is needed to improve alternative adjunctive approaches, particularly in light of the poor attendance at behavioral therapy sessions in many trials. Additionally, future clinical trials investigating the value of the addition of behavioral therapy may benefit from consideration of high-risk groups, such as people with a poor early response to buprenorphine for whom there may be more opportunity for treatment improvement.

## Notes

Supplement.
Data Sharing Statement
