# Supplementary material for: Behavioral Therapy as an Adjunct to Buprenorphine Treatment for Opioid Use Disorder: A Secondary Analysis of 4 Randomized Clinical Trials
Source: JAMA Netw Open. 2025 Aug 20;8(8):e2528529. doi: 10.1001/jamanetworkopen.2025.28529 (PMC12368672; doi:10.1001/jamanetworkopen.2025.28529)
Supplement: Supplement. — Data Sharing Statement [file jamanetwopen-e2528529-s001.pdf]

Data Sharing Statement

McHugh. Behavioral Therapy as an Adjunct to Buprenorphine Treatment for Opioid Use Disorder. *JAMA Netw Open*. Published August 20, 2025.
doi:10.1001/jamanetworkopen.2025.28529

Data
Data available: No
